# Editorial: Plant molecular farming for biopharmaceutical production and beyond

**DOI:** 10.3389/fpls.2025.1765245

**Published:** 2026-01-05

**Authors:** Kevin Yueju Wang, Yongfeng Guo

**Affiliations:** 1 Division of Math and Natural Sciences, University of Pikeville, Pikeville, KY, United States; 2 Tobacco Research Institute, Chinese Academy of Agricultural Sciences, Qingdao, China

**Keywords:** biopharmaceuticals, CRISPR/Cas9, plant molecular farming, plant-biotechnology, PMF

Plant Molecular Farming (PMF) continues to advance as a multidisciplinary field at the intersection of biotechnology, agriculture, and therapeutic development, yet challenges such as limited expression efficiency, protein instability, and scalability continue to shape its translational landscape. Recent studies have shown promising advancements in plant genetic engineering and novel expression systems, which have significantly improved the yield, purity, and functionality of plant-derived pharmaceuticals (Eidenberger et al., 2023; Akher et al., 2025). However, there remains a need for comprehensive research to address regulatory, economic, and ethical considerations, as well as to explore the successful commercialization and global impact of these plant-made pharmaceuticals. The articles in this Research Topic illustrate how platforms including *Nicotiana benthamiana*, *Arabidopsis thaliana*, moss, and chloroplast-based systems are evolving into flexible and scalable biomanufacturing hosts for therapeutics, metabolic products, and nanomaterials.

Several studies across this Research Topic highlight the continual refinement of *N. benthamiana* as a host for improved recombinant protein production. Suppressing the defense-associated genes Nb-SABP2 and Nb-COI1 markedly increased transient expression efficiency by reducing immune responses that normally limit heterologous protein accumulation, offering a practical strategy for enhancing yield without affecting plant growth (Kopertekh). Yang et al. also characterized the papain-like cysteine protease (PLCP) gene family and identified specific proteases that degrade recombinant proteins, revealing targets whose suppression can further improve protein stability in planta. Additional refinement comes from Kaur et al. (2025) employed multiplex CRISPR/Cas9 editing to disrupt all seven core glycosyltransferase genes responsible for plant-specific N-glycan motifs, generating stable, Cas9-free *N. benthamiana* lines completely lacking α-1,3-fucosyltransferase and β-1,2-xylosyltransferase activities. These glycoengineered plants grew normally and formed a robust platform for producing biotherapeutics with human-compatible glycosylation profiles.

Glyco-optimization remains central to enabling therapeutic-grade protein production in plants. Göritzer et al. demonstrated that modifying amino acids adjacent to the conserved Asn297 site, particularly through the Y300L variant, markedly increases N-glycan occupancy in plant-produced IgG1 and enhances Fc receptor binding and thermal stability, thereby reducing functional differences between plant- and mammalian-derived antibodies. Moss systems offer an additional avenue for controlled glycan engineering. Jonner et al. showed that introducing *Spodoptera frugiperda* mannosidase III and hexosaminidase into *Physcomitrium patens*, together with promoter tuning and targeted subcellular localization, enables production of recombinant human lysosomal acid α-glucosidase (Repleva GAA, RPV-002) enriched with paucimannosidic (MM) glycans. The engineered moss lines retained normal growth and achieved up to 43.5% of MM glycans, offering a practical strategy for improving mannose receptor–mediated uptake of therapeutic proteins.

Several articles in this Topic highlight the production of functional therapeutic molecules in plant systems. Yu et al. demonstrated that expression of an epidermal growth factor (EGF)–transdermal peptide fusion in *A. thaliana* yields a recombinant protein capable of penetrating the skin, and topical application markedly improves skin barrier repair in mice, illustrating the potential of plants for dermatological biologics. Melendez et al. reported that *N. benthamiana* can rapidly produce a monoclonal antibody targeting the extracellular enveloped virion (EV) form of Monkeypox virus; the plant-derived antibody bound the MPXV A35 antigen and neutralized EV particles, demonstrating that plant-based systems can generate antibodies with functional antiviral activity against this clinically important virion form. Wang et al. also compared the expression of full-length and mature nattokinase, revealing that the full-length precursor induces rapid leaf necrosis, whereas the mature enzyme accumulates efficiently and retains strong fibrinolytic activities, supporting its feasibility as a plant-produced therapeutic enzyme.

Viral nanoparticle technologies represent another major thematic area in this Research Topic. Ljumović et al. developed an innovative foliar spray infection method for producing Tomato Bushy Stunt Virus (TBSV)-derived viral nanoparticles in *N. benthamiana*, offering a simple and scalable alternative to syringe or vacuum infiltration and demonstrating compatibility with vertical farming systems. This advance is paired with a detailed description of a GMP-compliant manufacturing facility for producing clinical-grade plant-made nanomaterials, which outlines upstream cultivation, downstream purification, and regulatory considerations and provides a practical blueprint for industrial-scale nanoparticle production Pivotto et al.Vater et al. also demonstrated that *N. benthamiana* can produce functional human Galectin-1 (hGAL1) with correct folding, glycan binding, and immunomodulatory activities, reinforcing the suitability of plant systems for generating active immunotherapeutic proteins.

The Research Topic features significant contributions in metabolic engineering and agricultural biotechnology. Gerasymenko et al. demonstrated that co-expression of specialized isoprenyl diphosphate synthases (IDSs) and substrate-producing enzymes in *N. benthamiana* enables efficient biosynthesis of irregular monoterpene malonyl glucosides that are otherwise difficult to obtain, illustrating the versatility of plant systems for generating structurally diverse metabolites with pharmaceutical potential. Qi et al. reviewed advances in RNA interference as a precise and environmentally sustainable strategy for crop protection, highlighting developments in delivery platforms, regulatory considerations, and pathways for integration into large-scale agricultural practice. Schubert et al. utilized multilayered zein-based protein bodies in *N. benthamiana* to encapsulate both wild-type and hypoallergenic parvalbumin, enhancing resistance to gastrointestinal digestion and enabling controlled release. These engineered protein bodies were efficiently internalized by intestinal epithelial cells, establishing a promising plant-derived bioencapsulation platform for oral administration.

Together, these studies reflect a field moving rapidly toward technical maturity, translational relevance, and manufacturing readiness. We thank all contributing authors and reviewers for advancing the scientific and practical foundations of this evolving field, and we hope this Research Topic will inspire continued innovation across molecular farming applications.
